# Targeting Scavenger Receptors in Inflammatory Disorders and Oxidative Stress

**DOI:** 10.3390/antiox11050936

**Published:** 2022-05-09

**Authors:** Govigerel Bayarsaikhan, Delger Bayarsaikhan, Jaewon Lee, Bonghee Lee

**Affiliations:** 1 Lee Gil Ya Cancer and Diabetes Institute, Gachon University, Incheon 406-840, Korea; govio888@gachon.ac.kr (G.B.); degii07@gachon.ac.kr (D.B.); jwlee169@gachon.ac.kr (J.L.); 2 Department of Anatomy and Cell Biology, Graduate School of Medicine, Gachon University, Incheon 405-760, Korea

**Keywords:** AGEs, AOPPs, ALEs, scavenger receptors, oxidative stress, inflammation, gene knockout

## Abstract

Oxidative stress and inflammation cannot be considered as diseases themselves; however, they are major risk factors for the development and progression of the pathogenesis underlying many illnesses, such as cancer, neurological disorders (including Alzheimer’s disease and Parkinson’s disease), autoimmune and metabolic disorders, etc. According to the results obtained from extensive studies, oxidative stress–induced biomolecules, such as advanced oxidation protein products, advanced glycation end products, and advanced lipoxidation end products, are critical for an accelerated level of inflammation and oxidative stress–induced cellular damage, as reflected in their strong affinity to a wide range of scavenger receptors. Based on the limitations of antioxidative and anti-inflammatory molecules in practical applications, targeting such interactions between harmful molecules and their cellular receptors/signaling with advances in gene engineering technology, such as CRISPR or TALEN, may prove to be a safe and effective alternative. In this review, we summarize the findings of recent studies focused on the deletion of scavenger receptors under oxidative stress as a development in the therapeutic approaches against the diseases linked to inflammation and the contribution of advanced glycation end products (AGEs), advanced lipid peroxidation products (ALEs), and advanced oxidation protein products (AOPPs).

## 1. Introduction

Oxidative stress is currently a widely accepted term that describes the imbalance between oxidants and antioxidants that can contribute to the onset of several diseases. Reactive free radicals are generated from both endogenous and exogeneous sources during physiological activities and are crucial for modulating cellular metabolism, survival, death, signaling, differentiation, and so forth. Unfortunately, a lack of antioxidative defense or a disproportionate production of free radicals transforms them into harmful compounds that damage cells and biological molecules, including DNA, proteins, carbohydrates, lipids, and nucleic acids, causing the organism to lose its normal functioning [1]. In a biological system, all these modifications are simultaneously taking place, and the outcomes are complicated. Thus, to distinguish between these modifications caused by oxidative stress, the three most commonly used biomarkers are: (i) advanced glycation end products (AGEs); (ii) advanced oxidation protein products (AOPPs); and (iii) advanced lipid peroxidation products (ALEs) [2]. 

Inflammation is a prime example of a pathogenic process of oxidative stress, and the simultaneous involvement of oxidative stress and inflammation is prevalent in a variety of chronic diseases, such as cancer, metabolic disorders (such as diabetes and thyroid dysfunction), aging, cardiovascular diseases, kidney illness, ophthalmic disorders, and liver diseases [3,4,5,6,7,8,9,10]. Inflammation is the immune system’s response to both endogenous and exogenous harmful stimuli. Exogenous inducers can include microbial and non-microbial invaders, and endogenous inducers are both infectious and non-infectious matters. For exogenic stimuli, a major mechanism is the recognition of either pathogen-associated molecular patterns (PAMPs) or phagosomal damage in macrophages caused by allergens, irritants, or indigestible foreign materials; whereas inflammations triggered by endogenic stimuli are dependent on signals released from dysfunctional cells and tissues [11]. Furthermore, there are both acute and chronic forms of inflammation, depending on the extent and degree of responses. The silent and prolonged process of chronic inflammation has proven to be one of the leading causes of non-communicable diseases [12]. In comparison, acute inflammation takes place over a short time with severe symptoms and can be managed in a short period of time; persistent or intense inflammatory responses, as well as the inability to resolve them, can turn them into chronic conditions [13,14]. In addition, acute inflammation is mostly initiated by the activation of resident dendritic cells, macrophages, Kupffer cells, histiocytes, and mastocytes bearing surface receptors for the recognition of the specific molecules associated with pathogens [15]. Upon the activation of these immune cells, inflammatory mediators, including fragments of biochemical cascade systems, cytokines, chemokines, vasoactive amines/peptides, lipid mediators or proteolytic enzymes, are released from resident cells or plasma proteins [16]. In these mediators, a wide range of pattern recognition receptors, such as toll-like receptors (TLRs), NOD-like receptors (NLRs), c-type lectin receptors (CLRs), and scavenger receptors (SRs) play a crucial role and induce innate immunity [17]. Among them, scavenger receptors should be considered for their harmful effects that contribute to inflammation with oxidative stress due to their strong binding affinity to AGEs, ALEs, and AOPPs. Current findings demonstrated that a total of 12 classes of scavenger receptors (A to L) are known, and of them, 11 are found in humans, all except SR-C. They are superfamily of membrane bound receptors known for their ability to bind a wide range of pathogen and danger associated molecules, scavenge modified lipoproteins, and interact with other receptors, such as TLRs during pathogenesis of inflammatory disorders [18,19]. 

Moreover, a growing body of knowledge indicates that oxidative stress is more harmful when coupled with pathogenetic factors, such as hyper-glycation of intra- and extracellular proteins, hypoxia, immune activation, and inflammatory reactions [20]. Despite the fact that antioxidants are known as the best treatment against oxidative stress–induced inflammation, their practical application encounters several difficulties, including their pro-oxidative activities and dosage-dependent detrimental consequences, as well as the great risk of side effects associated with commercial anti-inflammatory drugs. Furthermore, the underlying mechanism of these biocompounds involves a blocking of two main inflammatory pathways, including nuclear factor kappa B (NF-kB) and mitogen-activated protein kinases (MAPK) [21,22]. However, these transcription factors are strongly associated with an AGE–RAGE-like axis during the pathogenesis of inflammatory conditions due to the intracellular oxidative stress caused by AGE–RAGE-like systems [23]. Overall, the reports suggest that strategies targeting AGEs such as molecules and the knockout/knockdown of their receptors can be alternatives to antioxidant-based approaches for treating oxidative stress–induced inflammatory disorders. Therefore, to highlight the present findings in oxidative stress and inflammatory process–related diseases regarding the deletion of the interaction between AGEs, ALEs, AOPPs, and their binding receptors, the current review was compiled. 

## 2. Advanced Oxidation of Protein Products (AOPPs) 

### 2.1. Definition of AOPPs

AOPPs are uremic toxins that were first described by Witko-Sarsat et al. as novel biomarkers for oxidized proteins in 1996 [24]. Structurally, AOPPs are heterogenous adducts that share similar characteristics with a wide range of chromophores, such as carbonyls, pentosidine, dityrosine, and disulfides. They are mostly found in plasma proteins, including albumin, globulin, fibrinogen, and lipoproteins, due to their exposure to oxidative stress, particularly chlorinated oxidants. Chlorinated oxidants, including hypochlorous acids, are formed in vivo through myeloperoxidase (MPO) enzymes that are expressed on immune cells [25]. Furthermore, MPO-chlorinated oxidant systems are activated in inflammatory conditions and play crucial roles as inflammatory mediators in inflammations, as well as in the generation of AOPPs [26]. However, a growing body of evidence has demonstrated that AOPPs accelerate the pathogenesis of a wide range of diseases by promoting oxidative stress and inflammatory responses, as exemplified by osteoarthritis, neuroinflammation, age-related bone loss, delirium, and organ damage caused by inflammation [27,28,29,30,31,32].

### 2.2. SRs That Recognize AOPPs in Inflammatory Conditions 

Studies have proven that AOPPs trigger their pro-inflammatory signals by binding to their cellular receptors. Among the early known receptors, AOPPs have demonstrated a strong affinity for the class B type I (SR-BI) receptor, which has been shown to bind high-density lipoproteins and is generally expressed on steroidogenic cells, hepatocytes, and macrophages [33,34]. Based on the conclusions of the authors, AOPPs act as strong pro-inflammatory mediators that damage high-density lipoprotein metabolisms and contribute to cardiovascular disorders by blocking their SR-BI receptors in vivo and in vitro. Furthermore, chronic inflammatory disease in blood vessels, such as atherosclerosis, is accelerated by the involvement of AOPPs in conjunction with inflammation and oxidative stress by promoting the formation of atherosclerotic plaques caused by an inhibition of the SR-BI receptor. However, AOPPs are not only metabolic disturbances, but are also a source for reactive oxygen species (ROS) and inflammatory mediators due to the potential to generate oxidative stress in a wide range of immune cells, including neutrophils, monocytes, and phagocytic cells [35]. Monocytes and macrophages are critical effectors and regulators of inflammation and the innate immune response. The macrophages play a crucial role in driving the fate of inflamed tissue and organs due to their multifunctional roles. Generally, macrophages are in two distinct subpopulations, including classically activated macrophages, or M1, and alternatively activated macrophages, or M2. Although M1 and M2 macrophages demonstrate positive and negative immune responses against inflammation (for example, M1 macrophages are responsible for secreting pro-inflammatory cytokines and chemokines, whereas M2 macrophages show anti-inflammatory effects), both are involved in the elimination of pathogens and the renovation of post-inflammation injuries [36,37]. 

In addition to SR-BI, AOPPs reveal a high affinity to CD36 (SR-B2), which is a membrane receptor protein with multifunctional immune–metabolic activities to mediate immunological recognition, inflammation, and cellular metabolic processes [38]. It was found that AOPPs induce arrest in the G1 phase of intestinal epithelial cells (IECs) through collective interaction with RAGE and CD36 receptors, which further leads to the phosphorylation of the signaling of c-Jun-N-terminal kinase (JNK) and the activated expression of p27kip1 signaling [39]. Furthermore, the regenerative damage of IECs may result in an immune system weakness against bacterial attack and inflammatory responses, as IECs play a crucial role in protecting the human body from microbial infections, and eventually inflammations [40]. Additionally, it is known that the toxicity of a system compromised by AOPPs and CD36 distresses the tubular epithelial cells, which play an important role in the response to inflammatory mediators during the pathogenesis of inflammatory diseases such as chronic kidney damage and ischemic heart diseases [41,42]. Moreover, it is known that AOPPs bind to receptors for advanced glycation end products, or RAGEs, to activate inflammation-mediating pathways. However, RAGE is known as receptor for AGEs, the high affinity of AOPPs with RAGEs can be emphasized by several factors with reference to AGEs, such as a close structural resemblance and comparable physicochemical characteristics, formation pathway (i.e., oxidation or cross-linking) accumulation, and carriage in identical proteins with a slow turnover rate in tissue and plasma [43]. RAGEs are member of the immunoglobulin superfamily and structurally compromised from two constant domains and a variable domain. Due to the presence of these multi domains, RAGEs show universal binding affinity to a wide range of ligands, regardless of their pattern, such as damage-associated molecular patterns or pathogen-associated molecular patterns from AGEs, AOPPs, and others (i.e., HMGB1, S100, and LPS). RAGE is expressed as several isoforms, including full length RAGE, N-truncated RAGE, soluble RAGE (sRAGE), and dominant negative RAGE. RAGEs deficient in N-terminal signal sequences, including N-truncated RAGEs, are incompetent to bind with proteins altered by oxidation, including AGEs, ALEs, and AOPPs, whereas full – length RAGEs can act as receptors for them to induce cellular signaling. In contrast, sequences encoding the transmembrane and intracytoplasmic domains are absent in the soluble form of RAGE or C-truncated RAGEs, thus they tend to be circulated in the blood stream and show strong binding affinity to AGEs – like molecules. In a recent study, it was found that levels of sRAGE and cell surface RAGE on peripheral blood mononuclear cells are negatively correlated in healthy human subjects. Functionally, sRAGE is a natural antagonist for AGEs mediated RAGE activation, and there are numerous studies reported benefits of antagonist effects of sRAGE in inflammatory conditions. Furthermore, compared to other forms, physiological role of full—length RAGEs are well known. For example, the axis formed with their ligands mediates various downstream signaling cascades associated with oxidative stress, inflammation, and cellular hemostasis [44,45,46,47,48,49]. However, RAGE is weakly expressed in most adult tissues under normal conditions, but their ligation leads to over expression of RAGEs, as well as accelerated progress of inflammation, and eventually the generation of inflammatory diseases such as diabetes, cardiovascular diseases, cancer, and neurodegenerative conditions via activation of NADPH oxidase and cellular ROS generation [50].

The AOPP–RAGE axis activates a wide range of pathways associated with oxidative stress. However, it is known that RAGE–AOPP bindings are involved in the progression of inflammatory diseases, but the underlying mechanism is quite complicated and requires comprehensive investigation for the development of therapeutic strategies to remedy or eliminate inflammatory responses. Regarding the mechanism, AOPP binding with RAGE is relatively well known in uremic diseases compared with others. For example, it was reported that AOPPs are upregulated with Wnt/β-catenin, which is a regulator of inflammation progression, to promote the damage of podocytes through various pathways, including the RAGE-dependent activation of nicotinamide adenine dinucleotide phosphate (NADPH) oxidase and factor-kB activation [51,52]. In addition to accumulation in organ tissues, AOPPs are also accumulated in the plasma of patients with diabetes and systemic inflammatory disorders, such as rheumatoid arthritis (RA). Accumulated AOPPs can then interact with RAGE to induce cell apoptosis during inflammation-induced diseases. This hypothesis was supported by findings obtained from Wu et al. In this work, the authors found that the exposure of chondrocytes to AOPPs results in the activation of NADPH oxidase and redox-dependent intrinsic cell death with the presence of RAGE [53]. Along with these results, a recent study discovered the possible mechanism underlying the involvement of RAGE in AOPP-induced inflammation. Yang et al. conducted in vitro experiments to induce AOPP stress in fibroblast-like synoviocytes, which play a crucial role in the pathogenesis of RA. They confirmed the overexpression of inflammatory cytokines, such as IL-6, INF-a, MMP3, and MMP13, followed by the activation of the NF-kB pathway due to the interaction between AOPPs and RAGE [54]. In 2013, Valente et al. applied a knockdown technique to silence the CD36 and RAGE receptors in mice, highlighting the important role of RAGE-AOPPs in cardiomyocyte deaths, with the CD36 knockdown not showing a significant difference for cell death under AOPP-induced stress. In this study, several proteins and pathways were reported for their promoting activity in the apoptosis of cardiomyocytes caused by AOPP–RAGE interactions, with the described candidates being Act1, Nox2/Rac1, and JNK [55].

Taken together, the findings indicate that AOPPs are capable of interfacing with several receptors on the cell surface, and that these interactions activate a wide range of pathways linked to oxidative stress, the release of inflammatory cytokines, and the development of pathogenesis mediated by both oxidative stress and inflammation. 

## 3. Advanced Glycation End Products (AGEs) 

### 3.1. Definition of AGEs

AGEs are generated through a non-enzymatic reaction between reducing sugar and the primary amine group of proteins, and as a result, unstable Amadori products are generated. Due to their reactivity, these products further undergo different modifications, including oxidation, cross-linking, dehydration, and cyclization, until the generation of irreversible final products, known as AGEs [56]. Regarding the reaction phases, glycation products can be categorized into three groups, those generated from: (i) early-stage reactions (i.e., Schiff bases); (ii) intermediate or glycoxidation products (i.e., Amadori degradation products); and (iii) terminal stage reactions (AGEs) [57]. Structurally, AGEs can be found in both aromatic and aliphatic forms, and among them, well known members are carboxymethyl-lysine (CML), carboxyethyl-lysine, glyoxal hydroimidazolone-1, methylglyoxal-derived lysine dimer, pentosidine, *3*-Deoxyglucosone-hydroimidazolone 1, pyrraline, imidazolone, methylglyoxal hydroimidazolone-1, and argpyrimidine [58]. Interestingly, AGEs are not only generated through non-enzymatic reaction, but can also be synthesized and secreted by certain types of immune cells. An example includes microglia, the only constituent of the macrophage population of the central nervous system. It was found that activated microglia produce albumin-linked AGEs, which may further interact with their receptor, RAGE, to activate neuroinflammation via microglial M1/M2 polarization [59,60].

As reviewed by Schmidt and colleagues, AGE toxicities are described in three distinct pathways, including: (i) accumulation in tissues and vessel walls, (ii) in situ glycation, and (iii) binding with receptors on the cell surface [61]. Accordingly, AGEs can be accumulated as an extracellular matrix, and an increased level of AGEs leads to the development of various inflammation-linked diseases, such as atherosclerosis, microvascular disorders, acute myocardial infarction, and diabetes [62,63]. AGEs could cause these issues by modifying protein structures and functions with the aid of cross-linking and altered hydrophobicity or binding to cellular immunoglobulin proteins located on the cell surface [64]. 

### 3.2. SRs That Recognize AGEs in Inflammatory Conditions 

The cell–AGE interface is advanced by the involvement of a wide range of receptors, such as RAGE, lactoferrin-like polypeptide (LF-L), toll-like receptor 4, and the scavenger receptors (SR-A, SR-B, FEEL-1, FEEL-2, CD36, LOX-1) of cells [65,66,67,68]. Among them, RAGE is known for its inflammation-triggering activity, whereas others are mostly supporting the degradation of AGEs [69]. Therefore, the scavenger receptors for AGE can possibly be classified into two groups—inflammation promoters and suppressors—with regard to their inflammatory response. The inflammation-enhancing activity of the AGE/RAGE system has been reported in various immune cells. An example includes dendritic cells, which are known for their central role in initiating antigen-specific adaptive immune responses. Recently, it was found that, compared with glucose-driven AGEs, those generated by fructose tend to severely activate dendritic cells through their RAGE-signaling pathways and yield various pro-inflammatory molecules, such as IL-6 and IL-1b [70]. The number of activated immune cells tends to increase during the progression of inflammation, and the pathogenesis of inflammation also leads to an increase in production of oxidants and AGEs, which can be disabled by targeting their source, including enzymes and receptors [71]. Among the immune cells that contribute to inflammation, macrophages are relatively well-known cells due to their capacity for generating AGEs, leading to the accelerated pathogenesis of AGEs-related diseases [72]. A wide range of molecules have been tested for their anti-inflammatory activities with regard to their blocking activity for AGE–RAGE interaction-induced inflammation using macrophage models. Recently, Yu et al. found that the polyphenolic antioxidant compound 4′-methoxyresveratrol is effective for blocking the activation of RAGE-induced inflammation reactions in macrophages via various pathways, including (i) blocking RAGE-mediated inflammasome pathways by suppressing the gene activation of pro-inflammatory enzymes and cytokines; (ii) showing antioxidant effects to inhibit NADPH oxidase and reducing the generation of toxic oxidative products, as well as AOPPs; and (iii) diminishing the overexpression of AGE-stimulated RAGE [73]. As a result of the initial activation of RAGE mediated by AGEs—like molecules, RAGE is stimulated through the NF-kB pathway, resulting in positive feedback loop [74]. 

In addition, it is known that AGE–RAGE interactions lead to the accumulation of fat in the liver, leading to various complications, such as inflammation and insulin resistance. Accordingly, it was found that elevated levels of AGEs are reversibly correlated with the anti-inflammatory activity of M2 macrophages, and positively linked to the pro-inflammatory response of M1 macrophages. Interestingly, a depletion in RAGE via the presence of sRAGE, which is known as a competitor to RAGE, leads to reduced levels of inflammation, macrophage phagocytosis, and the clearance of apoptotic cells in rat models [75]. Despite the RAGE knockout strategy, there are several AGE inhibitors, including alagebrium, pyridoxamine, aminoguanidine, and benfotiamine, which have been reported for their inhibiting activity against the harmful effects of AGE–RAGE interactions. Furthermore, a study performed on diabetic RAGE apoE double-knockout mice models showed that AGE-suppressing molecules may have the ability to prevent RAGE activation and block the expression of MCP-1, ICAM-1, and the macrophage marker CD11b, leading to decreases in macrophage accumulation, glomerular fibrogenesis, and cortical inflammation [76]. Similar results were observed by another research group, in which the authors suggested that pyridoxamine is capable of treating adipocyte-induced inflammation by suppressing M1 polarization as well as AGE–RAGE interactions in obesity model rats [77]. Furthermore, the AGE–RAGE axis–involved oxidation system was reduced along with the suppression of NADPH oxidase activity, the expression of inflammatory proteins, and macrophage accumulation due to the introduction of RAGE aptamers as an alternative option for AGE inhibitors [78]. Unfortunately, during the pathogenesis of certain diseases, including Alzheimer’s disease, the accumulation of AGE in cells or tissues tends to increase significantly, resulting in a burden of oxidative stress or apoptosis. As summarized by Srikanth et al., these conditions can be cleared by the application of therapeutic strategies targeting membrane-permeable antioxidants and AGE-inhibiting molecules by blocking AGE–RAGE-induced inflammation or the transportation of harmful molecules [79]. In a recent study of macrophages, Jahan and Cloudhary (2021) highlighted the suppressing activity of gliclazide (antidiabetic drug) against the M1 macrophage while promoting M2 macrophage phenotypes, which leads to a reduction in intracellular oxidative stress through the downregulation of AGE/RAGE signaling, as well as the interaction between Toll-like receptors and NF-kB complexes activated by reactive oxygen species [80].

There are two discrete populations that constitute the macrophages of the liver, including Kupffer cells and monocyte-derived macrophages [81]. These hepatic macrophages are responsible for maintaining the hemostasis of the liver; moreover, during inflammation, macrophages perform triple roles, such as antigen presentation, phagocytosis, and immunomodulation to drive pathways toward the instigation, progress, and fate of inflammation [82]. Growing bodies of evidence have demonstrated that exposure of AGEs to hepatocyte macrophages leads to accelerated cell proliferation, oxidative stress, and liver damage through the generation of an AGE–RAGE bond on macrophage surfaces [83]. However, the study results suggest that the hepatocyte toxicity of AGEs is less severe than for the AGE–RAGE system; however, interestingly, Bijnen et al. (2018) found that RAGE deficiency showed no effect on the pathogenesis of non-alcoholic steatohepatitis and atherosclerosis in hyperlipidemic/Ldlr knockout mice. Both disease models are inflammatory disorders generated by a Western-type diet containing a wide source of AGEs, including fats, sugars, and proteins [84,85]. In addition, their inflammation-inducing effects via AGE-induced RAGE activation were reported in numerous inflammation and inflammation-associated conditions, such as cardiac inflammation, cardiomyopathy macrophage activation, polycystic ovary syndrome, and diabetic nephropathy [86,87,88,89]. The considerable reduction in inflammation that is caused by the RAGE knockout strategy can be explained by the ability of AGEs to bind to a wide range of anti-inflammatory receptors, regardless of RAGE activation. This suggests that, in contrast to inflammation-suppressing receptors, AGEs show a significantly strong affinity to inflammation-activating receptors. In addition, the involvement of increased levels of AGEs in the pathogenesis of a wide range of inflammatory diseases has been proven in numerous studies, showing the application benefits of therapeutic approaches targeted at destroying the accumulation of AGEs against these inflammatory diseases.

Furthermore, AGEs interact with RAGE expressing on the mast cells known for their effector role in acute inflammatory responses. Mastocytes are a subset of white blood cells located in connective tissues, and their accumulation in inflammation sites was first identified by Ehrlich in 1879 [90]. Moreover, recent studies have found that the AGE–RAGE axis on mastocytes leads to increases in inflammation. For example, the AGE-dependent contribution of gut mast cells and their secreting histamine molecules was determined for inflammation after stroke [91]. According to the study, a number of mast cells and the activation of histamine receptors were observed in the gut inflammation area post-stroke, along with an increased level of pro-inflammatory cytokines, including IL-6, TNF-α, and G-CSF, as well as changes in the bacterial component of the gut. In another study, it was found that the AGE–RAGE-dependent activation of mast cells leads to low grade or chronic inflammation and an increased generation of reactive oxygen species. Interestingly, they analyzed the kinetics between the interaction of AGEs and mast cells with respect to the time required for the activation of RAGE. The results demonstrated that the first signal is observed in the first 20 s, and stabilized at 180 min upon AGE exposure. However, it was affected by presence of anti-RAGE antibodies, intracellular calcium, G(i) proteins, caffeine, and calcium chelator BAPTA-AM [92]. Calcium ion channels are one of the main regulators for the exocytosis of cells and play a decisive role in neurotoxicity and inflammations. For instance, due to the similarity of their charges, Mg^2+^ ions are capable of blocking their channels, and the strategy results in a reduced secretion of inflammatory and neurotoxic molecules in microglial and THP-1 cells [93]. In addition, it is known that immune cells express histamine to selectively activate effector cells to combat inflammation [94].

## 4. Advanced Lipoxidation End Products (ALEs)

### 4.1. Definition of ALEs

Lipids are diverse organic compounds, and regarding their chemical structure, there are eight groups of lipids, including fatty acyls, sphingolipids, saccharolipids, polyketides, sterols, prenols, glycerolipids, and glycerophospholipids [95]. They are a vital component of distinct biochemical reactions for energy storing, signaling, and the production of vital biological components, such as hormones and vitamins. However, the presence of oxidative stress leads to the degradation of lipid molecules and generates pro-oxidant carbonyls, such as lipid aldehydes and esters. Due to their stability, these reactive carbonyls potentially react with the nucleophilic residues of intra and extracellular macromolecules non-enzymatically, forming advanced lipoxidation products [96]. As both ALEs and AGEs are generated by the non-enzymatic modification/oxidation of biomacromolecules, they may share the same chemical structures and thus co-contribute to disease development [97]. An example includes glyoxal, which can be produced by either Schiff base fragmentation or lipid oxidation pathways, and further reacts with biomolecules to generate CML-like molecules that are known to be present in both ALEs and AGEs [98]. Altogether, this suggests that ALEs share common receptors with AGEs, and advanced instrumental analysis shows strong evidence of that. 

### 4.2. SRs That Recognize ALEs in Inflammatory Conditions 

It is recognized that ALEs show a strong binding affinity to receptors for AGEs, such as RAGE, galectin-3, and class A and B scavenger receptors. However, regarding the inflammatory role of ALE–receptor interactions, there is limited information, and the most studied receptor is RAGE. Interestingly, Mol et al. (2019) found that ALEs bind to RAGE in their neutral state under physiological environments. This interaction may lead to a disruption of the surface charge of the proteins, and unremittingly increase the electrostatic affinity of modified proteins with RAGE due to the release of negatively charged carboxylate groups on adducts [99]. 

The toxicity of the AGE–RAGE axis is known in a varied range of human and animal cells as a triggering factor for the activation of oxidative stress–inducing pathways and the expression of inflammatory genes, as discussed above. Accordingly, some studies have focused on revealing the toxic effects of the AGE–RAGE system on human cells, including monocytes under hyperglycemia conditions. For example, Shanmugam et al. reported that ALEs have the potential to lead to oxidative stress, as well as the upregulated expression of pro-inflammatory genes through the activation of NF-kB pathways in monocytes, which are known for their regulator and effector roles in inflammation. Among the AGE–RAGE-induced inflammatory cytokines, such as IP-10, COX-2, MCP-1, IL-6, IL-8, and several integrins, MDA-Lys specific inflammation marker, including eotaxin, was reported in this study [100]. 

Among the disorders affected by the interaction between ALEs and receptors is vascular calcification. However, inflammation plays an important role in the development and deposition of calcium due to the propagation reactions triggered by the pro-inflammatory stimuli response of macrophages and vascular smooth muscle cells. The fate of calcification is determined as either microcalcification or macrocalcification, depending on which receptor is activated. For example, whereas the interaction of infiltration with pro-inflammatory stimuli, including RAGE, AGEs, and ALEs, leads to microcalcification caused by inflammation reaction cycles, galectin-3 activation results in macrocalcification with stable plaque [101]. Further studies found that the lipid-induced activation of receptors leads to pro-inflammatory signaling pathways. Interestingly, compared with RAGE and scavenger receptors, galectin-3 displays a protective role against inflammatory conditions triggered by ALEs, including glomerular injury and atherosclerosis [102,103]. Moreover, glomerular damage is one of the most frequently reported injuries, due to oxidative stress and the accumulation of AGEs and ALEs. Thus, it may be considered as a definitive model for studies regarding the interaction of AGEs/ALEs with their receptors and their contribution to disease development. Along the same lines, Iacobni et al. (2005) generated glomerular lesions on a galectin-3 knockout mice model under hyperglycemic and oxidative stress conditions. They observed an incredibly high degree of age-dependent glomerular accumulation of toxic agents, including AGEs, ALEs, and reactive carbonyl species, as well as the activation of the NF-kB pathway in galectin-3 knockout subjects at variance with wild-type animals [104]. In addition to kidney damage, the involvement of galectin-3 receptors in ALE-mediated non-alcoholic steatohepatitis was reported, as the model can result from oxidative stress and fat accumulation [105]. It was demonstrated that the expression of galectin-3 is boosted up to 10 times, whereas the expression of RAGE increases approximately two times due to the prolonged intake of atherogenic diets in wild-type mice. In addition, the silencing of galectin-3 in the ALE-augmented liver area resulted in remarkable decreases in the activation degree of RAGE and, in contrast, their presence was correlated with increased levels of inflammatory indicators, such as the activation of immune cells, including monocytes, macrophages, and lymphocytes. Although expressions of pro-inflammatory cytokines comprising CXCR3, MCP-1, TNF-a, IFN-c, IL-4, IL-6, and IL10 were significantly increased due to the stimulation of the NF-kB/p65 and Th1/M1 pathways in wild-type mice exposed to high levels of ALEs, the ablation of the galectin-3 receptor showed suppressing activity against the expression of these genes. The authors explained the underlying mechanism as the absence of receptors for ALEs in liver endothelial cells leading to a decreased accumulation and uptake of ALEs and the further activation of other receptors or the initiation of inflammatory reactions [105]. This perhaps demonstrate that the binding affinity of ALEs towards galectin-3 is stronger than that of RAGE in hepatic environments, further driving the stimulation of RAGE and other receptors involved in ALE-mediated hepatic inflammation. 

Instead of silencing or knocking out receptors, antioxidant-based strategies against the accumulation of AGEs, ALEs, and AOPPs have been studied. In a recent study, Karasu’s group demonstrated that cysteine-containing antioxidants may have therapeutic effects against the pathogenesis of osteoarthritis through their suppressing activity on oxidative stress, the accumulation of ALEs and AGEs, the expression of some pro-inflammatory cytokines, and the activation of certain receptors, including RAGE and TLR-4 [106]. However, it was demonstrated that oxidized LDL dependent activation of TLR-4 upregulates TLR-4 as one of the pattern recognition receptors, and it is known for its expression on myeloid cells, including erythrocytes, granulocytes, and macrophages. There are numerous studies proving that these receptors interact with oxidized low-density lipoproteins (LDL) to induce inflammatory reactions during atherosclerosis. Moreover, it is found that TLR4 is time and dose dependently expressed by smooth muscle cells in response to exposure to oxidized LDL and is implicated in the generation of proinflammatory molecules (i.e., IL-1β, TNF-α, MCP-1, and MMP-2), and activation of NF-kb/p38 pathways in both in vitro and in vivo experiments [107]. In a recent study, accumulation of ROS and NOX2 expression downregulates Sirt1, Sirt3, and Mnsod [108]. 

**Figure 1:**
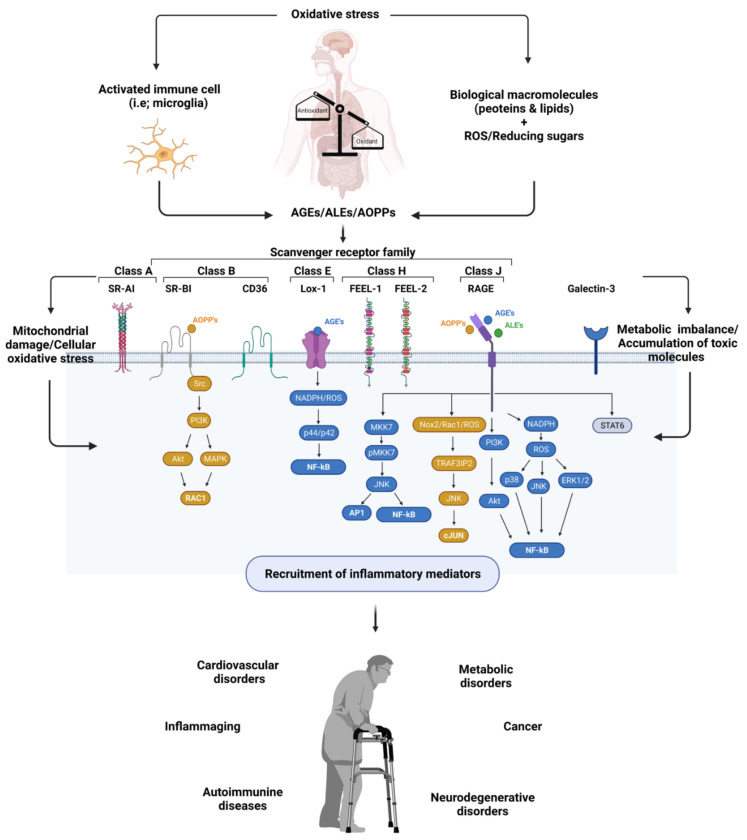
Exemplified figure for generation of inflammatory diseases with involvement of scavenger receptors and oxidative stress induced molecules, including AGEs, AOPPs, and ALEs; visualized using Biorender software [109].

Altogether, the accumulation of AGEs, ALEs, and AOPPs is highly correlated with the pathogenesis of inflammation and inflammation-induced disorders (Figure 1). Here, their interaction with cellular receptors plays an important role in their toxicity and is considered as one of the attractive points for researchers. 

## 5. Ablation of Receptors for AGEs, ALEs, and AOPPs in Inflammation 

With the advances in gene engineering, driving the expression of any protein is becoming conceivable, and their varied application is being intensively studied, showing enormous benefits in a wide range of fields, including therapeutics, diagnosis, drug screening, the generation of in vivo and in vitro disease models, and agriculture [110,111]. Along with the advances in gene engineering technologies and discovery of therapeutic molecules, a wide range of studies have been targeted to eliminate interaction between AGEs/AOPPs/ALEs and their receptors in oxidative and inflammatory disorders (Table 1). Methods for ablation of SRs include either knockout of target genes using CRISPR/Cas9 system or integration of target constructs into embryonic stem cells using electroporation and knockdown using siRNA. When applying traditional methods, two types of vectors are used, namely replacement vectors designed to replacing target exons with positive and negative selection events and insertion vectors designed to disrupt genetic loci. Despite the fact that this technique can disrupt target genes efficiently, it is expensive, time consuming, and requires a lot of labor [112]. However, the present gene engineering platforms, such as CRISPR/Cas, have enabled not only a better understanding of gene roles, but also easier genetic manipulation tools, which are more cost efficient, more time efficient, and provide more precise results with less off-target effects [113]. Among the CRISPR systems, the most commonly used tool is the CRISPR/Cas9 system, which is composed of Cas9 nucleases guided by small RNA molecules to generate site directed DNA cleavage. Currently, a wide range of receptors, including RAGE, CD36, SR-BI, and SR-A1, were successfully knocked out by CRISPR/Cas9 on different models, such as laboratory animals and cell lines [114,115,116,117,118,119]. 

The toxic effects of RAGE and their ligands, such as AGEs and ALEs, are well known in a large number of diseases covering most of the organ systems in humans and animals. Consequently, it is targeted by studies aiming to develop therapeutic strategies against diseases mostly mediated by oxidative stress, hyperglycemia, and hyperlipidation. For example, recent studies on mice demonstrated that knocking out the RAGE gene can protect respiratory systems from inflammation induced by the pollutants associated with smoking. It was found that RAGE knockout resulted in the upregulation of 179 genes and the downregulation of 351 genes that are predominantly associated with immune inflammatory responses in lungs [124]. Furthermore, the results illustrated the RAGE-driven activation of the RAS and NF-kB signaling pathways, which are major players in inflammatory responses characterized by an abundant penetration of alveolar macrophages and neutrophils in the lung area, and the knockout of RAGE showed suppressing effects on the activation of these pathways [136,137]. In a recent study, Frimat et al. found that, compared with wild-type mice, RAGE knockout mice are less prone to inflammation and oxidative stress under a CML-enriched environment. Moreover, it is interesting to note that this research group also examined the correlation between the subsistence of RAGE and the expression of antioxidant genes, including superoxide dismutase 2 (SOD2), catalase (CAT), and heme oxygenases (HMOX), which possess protective roles against oxidative stress and inflammation. They found that the expression of the CAT gene was not significantly affected by either the presence of RAGE or the aging process, whereas the SOD2 and HMOX genes tend to be expressed more intensively in young or RAGE knockout mice. Furthermore, the authors suggested that the existence of RAGE leads to a higher degree of inflammation or inflammaging compared with those knocked out for RAGE through the activation of AKT and SIRT1-induced NF-kB signaling [119]. Similarly, the deletion of RAGE on stromal cells showed that it is a major player in the pathogenesis of airway inflammation and encourages the signaling of type 2 inflammatory cytokines, such as IL-13 and IL-4 via inhibiting STAT6 activation [138]. 

In addition to respiratory organs, metabolic system organs, including the livers of mice, are also affected by RAGE with regard to inflammation and sepsis in both a DAMP (damage – associated molecular patterns)- and PAMP (pathogen-associated molecular patterns)-dependent manner [139]. Furthermore, as we know, ROS can be generated through either ex vivo or in vivo sources in living organisms. In the latter case, mitochondria are one of the major players, along with their contribution to the development of oxidative stress, in which the inner membrane uncoupling protein-2 (UCP2) has a significant role in balancing the production of ROS by mitochondria [140]. Indeed, the authors highlighted the contribution of dysfunctional mitochondria in the generation of oxidative stress, the accumulation of AGEs and ALEs, and the increased expression of RAGE and inflammation processes in the hepatic area or plasma of UCP2 mice models. They also emphasized that blocking the AGE–RAGE axis through RAGE complementary molecules holds great promise for reducing hepatic inflammation caused by damage in mitochondria [125]. However, mitochondria are highly susceptible to oxidative stress and the toxic effects of AGEs, ALEs, and AOPPS, despite their characteristic of being the main source for ROS [141]. Correspondingly, mitochondria dysfunction leads to cellular injury, apoptosis, the burden of oxidative stress, and a reduction in ATP production, thus showing a strong correlation with the pathogenesis of a wide range of diseases. For example, in recent study, Yu et al. found a strong correlation between a high fat diet and cardiac oxidative stress, in which the absence of RAGE showed a defensive role against oxidative stress induced by hyperglycemia and hyper-lipidation. The authors also stated that, due to RAGE knockout, the expression of the antioxidant enzyme SOD2 and the activation of sirtuin, which is responsible for the mitochondrial respiration process, were higher than those demonstrated in wild-type mice. However, it was also demonstrated that the accumulation of CML was dependent on the type of diet, without consideration of the contribution of RAGE. Interestingly, it was suggested that, although RAGE knockout has no effect on the expression of heart tissue inflammatory cytokines, it is capable of protecting against cardiac mitochondrial dysfunction via the upregulation of mitochondrial fission proteins and enhanced autophagic flux [126]. Similarly, numerous studies have proven that RAGE not only enhances inflammatory signaling, but also possesses protective roles through ligands, perhaps except AGEs. Along with this point, a wide range of studies have illustrated the benefits of RAGE in the resolution of inflammation processes through interaction with wide-ranging molecules, such as phosphatidylserine on macrophages to induce efferocytosis [127] and S100 proteins in corneal healing [128] and showing a protective role against tuberculosis; nevertheless, the underlying mechanism remains unclear [142]. 

Among the inflammatory processes, systemic inflammation is the most serious, with some of the syndromes being fatal. It is a process where the immune system is hyperactivated against harmful stressors, such as infection, surgery, acute inflammation, reperfusion, ischemia, and trauma. As a result, biological systems are reversibly and irreversibly damaged by the excessive secretion of pro-inflammatory cytokines and anti-inflammatory pathway homeostasis [143]. Examples of systemic inflammation-associated diseases include rheumatoid arthritis, sepsis, autoimmune diseases, and neurological disorders [144,145]. Indeed, the expression of RAGE on human adaptive immune cells has been reported in several studies with and without the commitment of their ligands, showing activating and modulating roles in immunity [146,147]. Altogether, this suggests that RAGE is an important contributor to the development of neurological and autoimmune diseases, regardless of their ligands, such as AGEs, ALEs, or AOPPs. However, whether RAGE is augmenting or mitigating the receptor depends on the interacting ligands, such as EN-RAGE (calcium-binding pro-inflammatory protein) or sRAGE (a soluble form of RAGE). Along with this point, Wu et al. found that the ratio of these two antagonistic binders of RAGE (EN-RAGE/sRAGE) is highly correlated with the pathogenesis of autoimmune hepatitis, showing the remarkable contribution of RAGE to the development of necroinflammation [148]. 

In the nervous system, the ablation of RAGE is described in several studies on inflammation-driving behaviors. Most of these studies suggested that knockout or genetic ablation of RAGE is capable of protecting AGE/ALE-induced damage and neurodegenerative diseases. For example, Zhang et al. demonstrated the protective role of the shortage in neuronal expression of RAGEs against synaptic injury caused by the toxicity of AGEs. Through the experiments performed in this study, where the perfusion of AGEs into brain slices of neuronal RAGE knockout mice was carried out under artificial cerebrospinal fluidic conditions, it was perceived that the AGE/RAGE system potentially diminishes synaptic plasticity and the augmented activation of MAPK P38 signals [149]. However, impaired synaptic plasticity and increased levels of AGEs are reported for numerous neurodegenerative disorders, including Alzheimer’s disease, and the link between central nervous system inflammation and systemic inflammation cannot be disregarded [150,151]. However, the effects of AGE/RAGE-like systems on the development and progression of neurodegenerative diseases are being focused on by researchers, and the underlying mechanism is still under debate. The main characterization of Alzheimer’s disease (AD) relies on an accumulation of the amyloid-β peptide in the brain; nevertheless, it was found that RAGE does not show any simulation on the phenotype of β-amyloidosis in mouse models of Alzheimer’s disease. Furthermore, the authors observed a strengthening in the activities of insulin-degrading enzymes in the brain, but no changes in amyloid-β increases or microglial activation due to the ablation of RAGE in one-and-a-half-year-old mice models. Although long-term studies may demonstrate some relationship between the absence of RAGE and the amelioration of AD, toxic ligands, such as AGEs and inflammatory reactions, are found during the pathogenesis of the disease [152]. Conversely, the inhibition of RAGE on non-neuronal cells, including microglia, showed a protective effect against ischemia-dependent synaptic dysfunction in an amyloid-enriched environment [153]. The background of the study considered the fact that RAGE expressed on both neuronal and non-neuronal cells under ischemic conditions, recognizing its ability to stimulate the deleterious effects of amyloid-β and cognitive impairment of AD patients. The in vitro experimental results proved that RAGE expression on microglial cells under ischemic conditions regulates both inflammatory pathways and synaptic functions through the simultaneous activation of the p38MAPK and JNK pathways. However, it was observed that RAGE/amyloid-β signaling in glial cells triggers synaptic depression through modulating activity for the secretion of IL-1β, a key mediator of inflammatory responses [153]. Furthermore, the excessive generation of ROS leads to hypertension, which is the main contributor to the development of cardiovascular disorders [154]. Interestingly, the relationship between hypertension and brain damage that is induced with an accumulation of amyloid-β was studied on RAGE knockout mouse models by a research group led by Lembo in 2013. They found that, during the pathogenesis of hypertension, AGE/RAGE activates in brain vasculature to enhance the accumulation of amyloid-β and triggers corresponding secondary difficulties, such as memory deterioration and cognitive impairments [155]. Nonetheless, memory impairment resulting from the interaction of RAGE with high mobility group box-1 or, conversely, the involvement of other receptors, such as amyloid-β receptors, in the pathogenesis of neurodegenerative disorders was reported [156,157]. This demonstrates the importance of considering not only the RAGE axis with AGEs/ALEs or AOPPs, but also their alignment with other ligands, and vice versa. 

Chronic inflammation, which comes along with the degradation of extracellular matrixes (i.e., loss of elastin and activation of matrix metalloproteinases, such as MMP-9 and MMP-2), leads to the development and progress of the pathogenesis of aneurysms. Zhang et al. determined a correlation between blocked RAGE and the pathogenesis of abdominal aortic aneurysm with regard to several factors, including: (i) MMP-9, a main mediator for extracellular degradation, is responsible for the regulation and control of inflammations; and (ii) the AGE/RAGE-induced expression of pro-inflammatory molecules, such as VCAM-1 and TNF-a, is followed by the accumulation of AGEs in vessel walls. It was reported that the prevalence of abdominal aortic aneurysm among the mice was reduced by up to three times due to RAGE knockout, and the underlying mechanism was explained by dose-dependent increases in MMP-9, along with increases in AGEs and signaling of the AGE/RAGE axis on macrophages [158]. 

Due to their central role in inflammatory diseases and their interaction with oxidized lipoproteins, scavenger receptors have been targeted by researchers over the last two decades. Along with these points, CD36 has shown strong evidence of involvement in the pathogenesis of inflammations triggered by oxidative stresses, and the SR-BI receptor is pursuing. CD36 is expressed on various cells, including monocytes, macrophages, adipocytes, and platelets, and is known as a pattern recognition receptor for modified LDL, thus playing a pivotal role in cellular lipid accumulation and inflammatory reactions [159]. Current results demonstrate that CD36 is responsible for NF-kB–mediated macrophage inflammatory responses that are initiated by mitochondrial metabolic reprogramming under the presence of oxidized low-density lipoproteins and the accumulation of long chain fatty acids [160]. Conversely, CD36 knockout mice fed with a high cholesterol diet demonstrated a reduction in oxidative stress, NF-kB activation, and a secretion of pro-inflammatory cytokines/chemokines, while showing increases in macrophages along with suppressed fibrogenesis and adipose tissue inflammation [161,162]. The upregulation of CD36 and the activation of Toll-like receptor 4 and NF-kB pathways showed positive correlation with the interaction between oxidized LDL and phosphatase receptor type O, or PTPRO. In addition, it was found that PTPRO knockout leads to decreases in ROS and MDA, as well as cell apoptosis [163]. Along with the previous findings demonstrating the major role of PTPRO in acute inflammation initiated by NF-kB–activated immune cells, study results show the importance of targeting PTPRO versus the pathogenesis of inflammatory disorders caused by oxidative stress combined with excess levels of glucose or lipids [164].

## 6. Conclusions

Current studies have proven that oxidative stress biomarkers, including AGEs, ALEs, and AOPPs, are heavily engaged in the pathogenesis of various inflammatory disorders, in which their interaction with scavenger receptors play an important role in the activation of inflammatory pathways or mitochondrial damage, and are driving homeostasis of cells and the pathogenesis of the diseases. In spite of the fact that the term “scavenger receptor” covers quite a number of different receptors, there is limited information available for SR-AGEs like systems regarding their contribution to inflammatory reactions under oxidative stress for each receptor. Furthermore, based on this perception, the most well- known receptors are RAGE, SR-AI, SR-BI, CD36, LOX-1, and galectin-3, which provides evidence for the necessity of conducting more comprehensive research on this subject to develop successful therapeutic approaches against inflammation and oxidative stress. Advances in gene engineering are being extensively employed in therapeutic approaches targeting the deletion of receptors, showing promising benefits for the effective and safe management of inflammation, both in vivo and in vitro. Furthermore, in contrast to antioxidant or anti-inflammatory molecule-based approaches against oxidative stress and inflammations, such genetic approaches may promote the natural secretion of certain antioxidant genes while solving several difficulties, such as the short lifetime of remedial molecules, dosage-sensitive effects, and drug-linked secondary effects.

## Figures and Tables

**Table 1:** Ablation of scavenger receptors of AGEs, AOPPs, and ALEs as therapeutic approaches for inflammatory disorders.

Ligand	Receptor	Condition	Up (↑) and Downregulated (↓) Molecules	Ref.
AGEs	RAGE	Renal aging	IL-6, TNF-α, VEGF, pS6RP, and pAKT (↓); Sod2, S6RP, and SIRT1 (↑)	[120]
		Alzheimer’s disease	iNOS, COX2, NLRP3,pMLC, and CD16/32 (↓)	[60]
		Neuroinflammation	iNOS, COX2, NLRP3,IL-6, TNF-α, pIkB, NF-kB, pp38, and GSK3β (↓)pERK and pJNK (no change)	[121]
		Diabetes mellitus	iNOS, IL-6, TNF-α, CD86, and NF-kB (↓)	[122]
		Non-alcoholic fatty liver disease and steatohepatitis	Nf-kB, NRF2, NLRP3, IL-1b, Glo-1, AGE-R1 (↓); Gal-3 (↑)	[123]
		CS-induced airway inflammation	IL-1β, IL-6, and TNF-α (↓)	[124]
		Liver failure	RAGE (↓)	[125]
		Myocardial dysfunction	TNF-a, IL-1b, and ICAM1 (↓); SIRT1, SIRT3, and VCAM-1 (↑)	[126]
		Inflammation	IL-10 (↑)	[127]
		Corneal wound	IL-1b, IL-6, and S100-A4 (↓)	[128]
		Diabetic retinopathy	pERK and VEGF (↓)	[129]
	SR-A	Diabetes mellitus	pERK (↓); pJNK, pIkBa, NF-kB, Ap-1, IL1-b, IL-6, IL-10, IL-12, TNF-α, MCP-1, VEGF (↑)	[130]
	CD36	Atherosclerosis in diabetes mellitus	Cdk5 (↑)	[131]
	Galectin-3	NFLD	PPARγ, PPARα (↑)	[132]
		Diabetic glomerulopathy	TGFβ, NF-kB (↑), MMP-2, MMP-14 (↓)	[105]
		Nonalcoholic steatohepatitis	LXR-α, LXR-β (↓); PPARγ, Abca-1, CD36 (↑)	[133]
ALEs	Galectin-3	Nonalcoholic steatohepatitis	TGF-β, NF-kB CXCR3, MCP-1, TNF-α, IL-4, IL-6, IL-10, IFN-γ, COX-2 (↓)PPARγ, PPARα (↑)	[134]
AOPPs	RAGE	Cardiomyocyte death	TRAF3IP2 (↓)	[55]
	CD36	Hepatic steatosis	IL-15, Cpt1, Fabp3, Fndc5 (↑)	[135]
